# Improved prognosis of advanced-stage extranodal NK/T-cell lymphoma: results of the NKEA-Next study

**DOI:** 10.1038/s41375-025-02527-4

**Published:** 2025-02-17

**Authors:** Ayumi Fujimoto, Kana Miyazaki, Kimikazu Yakushijin, Takahiro Fujino, Wataru Munakata, Yasuo Ejima, Dai Maruyama, Nobuko Kubota, Takeshi Maeda, Jun Takizawa, Nobuhiro Hiramoto, Masahiro Takeuchi, Rika Sakai, Noriko Fukuhara, Senzo Taguchi, Naoko Asano, Motoko Yamaguchi, Ritsuro Suzuki

**Affiliations:** 1 Division of Hematology and Oncology, Department of Internal Medicine, Faculty of Medicine, Shimane University, Izumo, Japan; 2 Department of Hematology and Oncology, Mie University Graduate School of Medicine, Tsu, Japan; 3 Division of Medical Oncology and Hematology, Department of Medicine, Kobe University Hospital, Kobe, Japan; 4 Division of Hematology and Oncology, Department of Medicine, Kyoto Prefectural University of Medicine, Kyoto, Japan; 5 Department of Hematology, National Cancer Center Hospital, Tokyo, Japan; 6 Department of Radiology, Dokkyo Medical University, Shimotsuga, Japan; 7 Department of Hematology Oncology, Cancer Institute Hospital, Japanese Foundation for Cancer Research, Tokyo, Japan; 8 Division of Hematology, Saitama Cancer Center, Ina, Japan; 9 Department of Hematology and Oncology, Kurashiki Central Hospital, Kurashiki, Japan; 10 Department of Hematology, Endocrinology and Metabolism, Faculty of Medicine, Niigata University, Niigata, Japan; 11 Department of Hematology, Kobe City Medical Center General Hospital, Kobe, Japan; 12 Division of Hematology-Oncology, Chiba Cancer Center, Chiba, Japan; 13 Department of Hematology and Medical Oncology, Kanagawa Cancer Center, Yokohama, Japan; 14 Department of Hematology, Tohoku University Hospital, Sendai, Japan; 15 Department of Radiation Oncology, Cancer Institute Hospital, Japanese Foundation for Cancer Research, Tokyo, Japan; 16 Department of Molecular Diagnostics, Nagano Prefectural Shinshu Medical Center, Suzaka, Japan; 17 Department of Hematological Malignancies, Mie University Graduate School of Medicine, Tsu, Japan

**Keywords:** Risk factors, Medical research

## Abstract

A retrospective study of extranodal natural killer/T-cell lymphoma (ENKL) patients diagnosed between 2014 and 2021 in Japan was conducted. Among 351 patients with sufficient data, 116 (33%) were in the advanced stage (5 in stage III and 111 in stage IV) at diagnosis, and were further analyzed. The median age was 60 years (range: 19–90), and 68 (59%) were male. Ninety-four (85%) of stage IV patients had two or more extranodal involvements. The most common first-line regimen was SMILE (steroid, methotrexate, ifosfamide, L-asparaginase, and etoposide; 52%). The 2-year overall survival (OS) for all patients was 38.5%, which was significantly improved after 2017 (25.2% for 2014–2017 vs. 50.7% for 2018–2021; *P* = 0.008). Patients treated with SMILE showed better OS than those treated with DeVIC or CHOP (2y-OS: 57.1%, 35.8%, and 0%, respectively; *P* < 0.001). The prognosis was significantly better in patients who received hematopoietic stem cell transplantation (HSCT) than in those who did not (2-year OS: 68.3% vs. 17.6%, *P* < 0.001). Multivariate analysis showed SMILE and HSCT were significant factors for OS. In conclusion, the prognosis of advanced-stage ENKL has improved in recent years. The L-asparaginase-containing chemotherapy and subsequent HSCT is considered the recommended strategy.

## Introduction

Extranodal natural killer/T-cell (NK/T-cell) lymphoma (ENKL) is one of the mature T/NK-cell neoplasms with a particularly high prevalence in East Asia and South America. ENKL is a distinct lymphoma subtype characterized by a strong association with the Epstein-Barr virus (EBV) for its pathophysiology, and predominant involvement of the nasal cavity and nasopharynx. While the majority of ENKL patients are confined to the region around the nose or upper aerodigestive tract, approximately 30% of them exhibit systemic involvement at the time of diagnosis [1].

Before the early 2000s, the prognosis of ENKL patients was poor, particularly for advanced-stage patients, with a median overall survival (OS) of less than 1 year [2–4]. This resulted from the majority of patients being treated with cyclophosphamide, doxorubicin, vincristine, and prednisone (CHOP)-like chemotherapy, similar to other B-cell or T-cell lymphoma patients. Because NK-cells, even normal NK-cells, express multi-drug resistance (MDR)-associated P-glycoprotein on their surface [5, 6], anthracycline-containing chemotherapies including CHOP, are less effective for ENKL patients. Subsequently, several chemotherapies, primarily including non-MDR-associated anti-cancer agents, have been developed for ENKL patients.

For limited-stage ENKL patients, concurrent chemoradiotherapies (CCRT), such as radiotherapy with a two-thirds dose of dexamethasone, etoposide, ifosfamide, and carboplatin (RT-2/3DeVIC), and radiotherapy with weekly cisplatin followed by etoposide, ifosfamide, cisplatin, and dexamethasone (CCRT-VIPD), were established as first-line treatments [7, 8]. For advanced-stage, relapsed, or refractory ENKL patients, L-asparaginase-containing and non-anthracycline-based chemotherapies, such as steroid, methotrexate, ifosfamide, L-asparaginase, and etoposide (SMILE), and modified SMILE using peg-asparaginase instead of L-asparaginase of SMILE, were established as first-line treatments [9, 10]. The prognosis for stage IV, relapsed, or refractory ENKL patients treated with SMILE was 55% at 1 year and was significantly better in patients who had undergone hematopoietic stem cell transplantation (HSCT) compared with those who had not [9].

The NKEA study, our previous retrospective analysis of 358 ENKL patients diagnosed between 2000 and 2013 in Japan, showed that the 5-year OS for limited-stage and advanced-stage ENKL patients were 68% and 24%, respectively [1]. These were higher than those obtained in our historical study [4]. The latest data on ENKL patients diagnosed in Japan after 2013 has not yet been collected and analyzed. Therefore, we subsequently conducted a nationwide study in Japan, the NKEA-Next study, to understand the current status of the most recently diagnosed ENKL patients. This study aimed to explore the trend in treatments, assess their response rates, and evaluate the latest outcomes of ENKL patients. Data from limited-stage patients are presented elsewhere (Miyazaki K, et al., in preparation), and herein, we present the results of advanced-stage ENKL patients.

## Methods

### Patient selection and study overview

This multicenter retrospective study of ENKL patients diagnosed between 2014 and 2021 was conducted to elucidate the current treatments and outcomes for ENKL in Japan (NKEA-Next project: UMIN 000046300). Data were collected on consecutive patients at each participating institute. The diagnosis of ENKL patients was initially performed by physicians and pathologists at each institute based on the 4^th^ or revised 4^th^ edition of the WHO classification [11]. In this study, the diagnosis of each patient was re-evaluated by investigators. Central pathological reviews by an expert hematopathologist (N.A.) were conducted for cases lacking sufficient information regarding ENKL diagnosis, including the immunohistochemistry results of CD3, cytotoxic molecules, and EBV-encoded RNA in situ hybridization.

A total of 368 ENKL patients were initially registered in our cohort. Patients diagnosed with other lymphoma subtypes by the central pathological review (*n* = 8), those with insufficient clinical data (*n* = 7), and duplicates (*n* = 2) were excluded. Consequently, 351 patients, including 235 limited-stage patients and 116 advanced-stage patients from 44 institutes in Japan, were eligible. In the present study, we focused on the analysis of 116 advanced-stage ENKL patients. Data were compared, as necessary, with our previous NKEA study [1], including 101 advanced-stage ENKL patients diagnosed between 2000 and 2013.

### Ethics approval and consent to participate

This study adhered to the Declaration of Helsinki and received approval from the institutional review board of Mie University (approval number: H2021-239) and each participating institute. All methods were performed in accordance with the Japanese Ethical Guidelines for Medical and Biological Research Involving Human Subjects. Informed consent was obtained in the form of opt-out since this is a retrospective study, which is in accordance with the Japanese guidelines and regulations. The study execution it was publicly disclosed at all participating institute websites, providing all patients with the opportunity to decline participation.

### Outcome definitions

The patient’s general condition at diagnosis was evaluated using the Eastern Cooperative Oncology Group Performance Status (ECOG-PS). The prognostic index of natural killer lymphoma (PINK) and PINK-E were evaluated as previously described [12]. Staging and treatment response was assessed according to standard criteria by physicians at each institution [13, 14]. OS was defined as the time from the date of diagnosis to the date of death by any cause. Surviving patients were censored at the last follow-up.

### Statistical analysis

Survival data were analyzed using the Kaplan-Meier method and compared using the log-rank test. When comparing the trend of chemotherapy regimens used as first-line treatment, data from the previously reported NKEA study were included [1]. To adjust the confounding factors of other variables for OS, univariate and multivariate analyses were performed using Cox’s proportional hazard regression model, and hazard ratios (HRs) were calculated in conjunction with the 95% confidence interval (CI). Two-sided *P*-values of less than 0.05 were considered significant. Analyses were performed using Stata version 14.0 (Stata Corporation, College Station, TX).

## Results

### Patient characteristics

The demographics of the 116 advanced-stage ENKL patients are presented in Table 1. The median age was 60 years (range: 19–90), and males accounted for 59% (*n* = 68) of the cohort. Fifty-two patients (45%) were diagnosed between 2014 and 2017, whereas 64 (55%) patients were diagnosed between 2018 and 2021. Forty patients (34%) had poor ECOG-PS higher than 1, and 62 (54%) had B symptoms at diagnosis. Among the 116 patients, 5 (4%) were in stage III, and 111 (96%) were in stage IV. Of the 111 stage-IV patients, 94 (85%) had two or more extranodal involvements at diagnosis, including the nasal and/or paranasal area (68%), bone and/or bone marrow (47%), skin (44%), lung (18%), liver (17%), spleen (14%), and central nervous system (10%). Seventy-three patients (63%) exhibited elevated levels of lactate dehydrogenase (LDH) exceeding the upper limit of normal (ULN), and 98 (84%) showed elevated serum soluble interleukin-2 receptor levels (sIL2-R) exceeding the ULN at each institute. Regarding the PINK, 20 patients (17%) were classified as intermediate-risk, with advanced stage being the solo risk factor (Table 1). Among the 73 patients tested for EBV-DNA in peripheral blood, 67 (92%) had detectable EBV-DNA. According to the PINK-E, 2 patients (3%) were classified as low-risk, 17 (23%) as intermediate-risk, and 54 (74%) as high-risk. The differences in the patient characteristics based on first-line treatment and HSCT status are listed in Table 1 and Supplemental Table S1, respectively.

**Table 1:** Characteristics of advanced-stage ENKL patients.

		ALL (*n* = 116)	SMILE (*n* = 55)	DeVIC (*n* = 31)	CHOP(-like) (*n* = 14)	*P*
		*N* (%)	*N* (%)	*N* (%)	*N* (%)	
Age	median, years (range)	60 (19–90)	51 (20–82)	67 (19–84)	65.5 (38–83)	
	≥60 years	58 (50)	15 (27)	19 (61)	9 (65)	0.002
Gender	Male	68 (50)	31 (56)	20 (65)	7 (50)	0.61
	Female	48 (41)	24 (44)	11 (35)	7 (50)	
Year at diagnosis	2014–2017	52 (45)	21 (38)	16 (52)	5 (36)	0.44
	2018–2021	64 (55)	34 (62)	15 (48)	9 (64)	
ECOG-PS > 1		40 (34)	13 (24)	11 (35)	6 (43)	0.26
B symptoms		62 (54)	32 (58)	15 (48)	6 (43)	0.50
Stage	III	5 (4)	3 (5)	1 (3)	1 (7)	0.84
	IV	111 (96)	52 (95)	30 (97)	13 (93)	
Extranodal site involvement ≥ 2		94 (85)	43 (81)	27 (90)	9 (75)	0.39
Bulky disease > 7 cm		19 (16)	8 (15)	5 (16)	3 (21)	0.80
Hb < 11 g/dl		28 (24)	9 (16)	10 (32)	3 (21)	0.23
Plt < 150 × 10^9^/L		34 (29)	10 (18)	13 (42)	3 (21)	0.06
CRP > 1 mg/dl		50 (43)	19 (35)	15 (48)	9 (64)	0.10
LDH > ULN		73 (63)	36 (65)	19 (61)	6 (43)	0.28
sIL-2R > ULN		98 (84)	48 (87)	25 (81)	13 (93)	0.61
EBV-DNA	Detectable^a^	67 (58)	39 (71)	20 (65)	5 (36)	0.12
	Undetectable	6 (5)	3 (5)	1 (3)	1 (7)	
	Not tested	43 (37)	13 (24)	10 (32)	8 (57)	
PINK	Intermediate (1)	20 (17)	13 (24)	5 (16)	1 (7)	0.38
	High (2–4)	96 (83)	42 (76)	26 (84)	13 (93)	
PINK-E	Low (1)	2 (2)	1 (2)	1 (3)	0 (0)	0.71
	Intermediate (2)	17 (15)	10 (18)	4 (13)	2 (14)	
	High (3–5)	54 (47)	31 (56)	16 (52)	4 (29)	
	Not evaluable	43 (37)	13 (24)	10 (32)	8 (57)	

ECOG, Eastern Clinical Oncology Group; PS, performance status; Hb, hemoglobin; PLT, platelet; CRP, C-reactive protein; LDH, lactate dehydrogenase; ULN, upper limit of normal; sIL-2R, soluble interleukin-2 receptor; EBV, Epstein-Barr virus; PINK, prognostic index of natural killer lymphoma.

^a^Only patients with EBV-DNA detectable in the peripheral blood.

### First-line treatment for advanced-stage ENKL patients

**Fig. 1:**
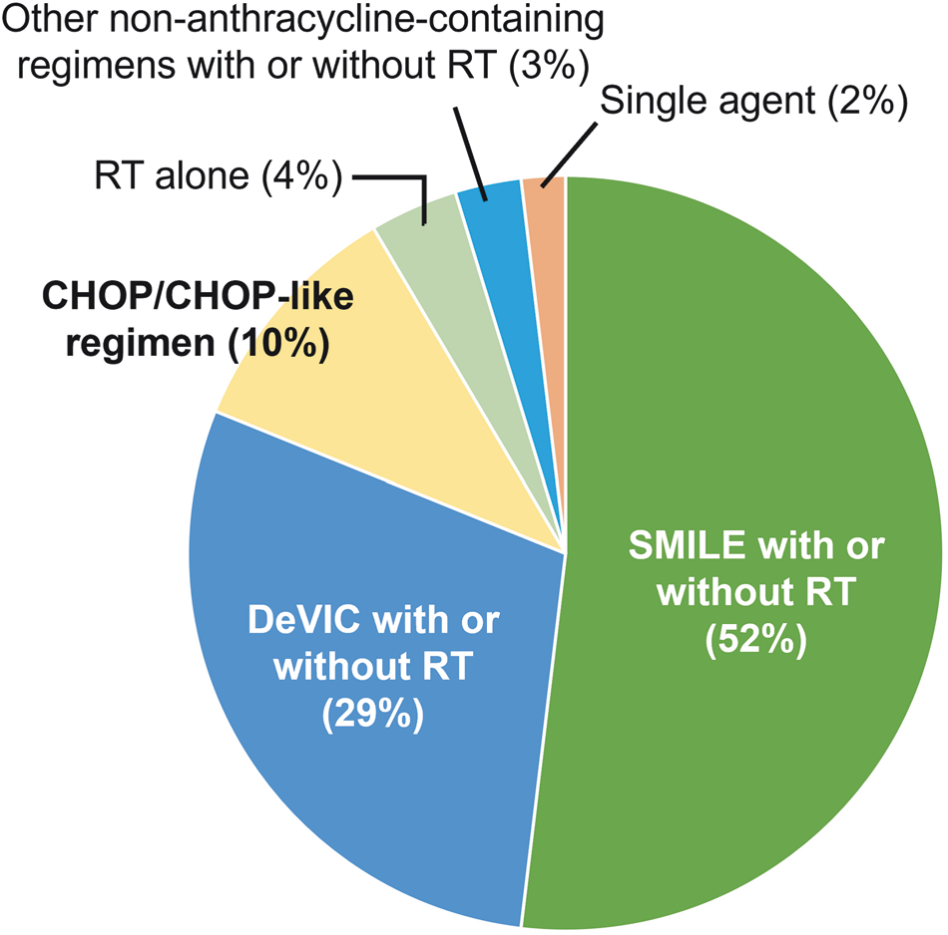
Proportions of first-line treatments. The most common first-line chemotherapy regimen for 106 patients with advanced-stage ENKL was SMILE (steroid, methotrexate, ifosfamide, L-asparaginase, and etoposide; n = 55, 52%) with or without radiotherapy (RT), followed by DeVIC (dexamethasone, etoposide, ifosfamide, and carboplatin; n = 31, 29%), CHOP (cyclophosphamide, doxorubicin, vincristine, and prednisone), including CHOP-like regimen (n = 11, 10%), and other non-anthracycline-containing regimens such as GDP (gemcitabine, dexamethasone, and cisplatin) and GCD (gemcitabine, carboplatin, and dexamethasone).

Among the 116 advanced-stage ENKL patients, 106 (91%) received treatment. Ten patients (9%) did not receive any treatment due to their poor general condition and intolerance to chemotherapy at diagnosis. Among the 106 treated patients, 86 (74%) received chemotherapy alone, 7 (6%) received CCRT, 9 (8%) received sequential chemoradiotherapy, and 4 (3%) received only radiotherapy (RT) (Supplementary Table S2). Among the 20 patients who received RT with or without chemotherapy, 16 patients (80%) received RT targeting the nasal cavity and/or sinuses, which were the largest site of disease. Of the remaining 4 patients, 1 received whole-brain RT for CNS involvement, 1 for the eyeball, 1 for a 10 cm lesion of testis, and 1 for a single subcutaneous lesion. Regarding the chemotherapy regimen with or without RT, the most common first-line chemotherapy regimen among the treated 106 patients was SMILE (*n* = 55, 52%), followed by DeVIC (*n* = 31, 29%), CHOP (including CHOP-like regimen) (*n* = 11, 10%), and other non-anthracycline-containing regimens such as GDP (gemcitabine, dexamethasone, and cisplatin) and GCD (gemcitabine, carboplatin, and dexamethasone) (Fig. 1 and Supplementary Table S2). Two patients received a non-SMILE regimen (both DeVIC) with L-asparaginase. The changing trends of chemotherapy are shown in Supplementary Fig. S1. The most commonly used chemotherapy regimen was CHOP between 2000 and 2004, followed by DeVIC between 2005 and 2009, but changed to SMILE between 2014 and 2021 (Supplementary Fig. S1).The median cycle of SMILE was 3 (range, 1–6 cycles), and 29 of the 55 patients treated with SMILE (53%) experienced at least one nonhematologic adverse event (AE) of grade 3 or higher, although the information regarding nonhematologic AEs in one patient was not available (Supplementary Table S3). Among the 55 patients treated with SMILE, 4 patients also received RT (Supplementary Table S2). The most common grade 3 AE was febrile neutropenia (13%), followed by liver dysfunction (11%), and anorexia and oral mucositis (7% each). One patient died after the initiation of SMILE due to worsening interstitial pneumonitis, which was previously diagnosed and was not considered to be related to SMILE chemotherapy.

**Fig. 2:**
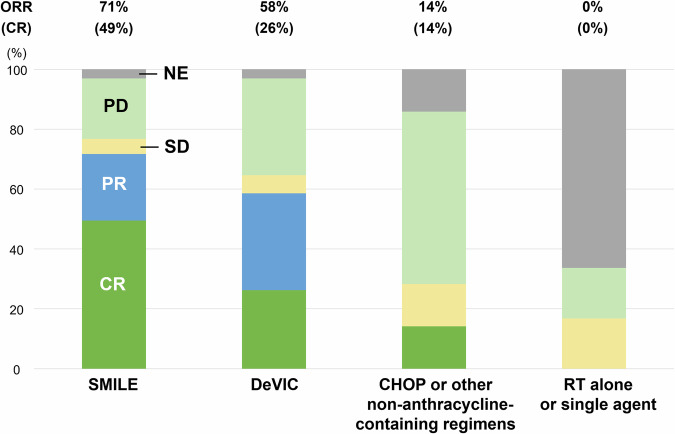
Response by the chemotherapeutic regimens. The overall response rate (ORR) of patients who received SMILE was 71%, and the complete response (CR) rate was 49%. The ORR of DeVIC, CHOP/other regimens, and radiotherapy (RT)/single-agent chemotherapy was 58%, 14%, and 0%, respectively. The CR rate of DeVIC and CHOP/other regimens was 26% and 14%, respectively.

The overall response rate (ORR) of patients who received SMILE was 71%, which was significantly higher than that of those treated with DeVIC (58%), or CHOP/other regimens (14%) (Fig. 2). The complete response (CR) rate was also the highest in patients treated with SMILE (49%), followed by DeVIC (26%), and CHOP/other regimens (14%). No patients who were treated with RT alone or single-agent chemotherapy achieved a partial response (PR) or CR. The proportion of patients who underwent subsequent HSCT was significantly higher in those treated with SMILE than in those treated with a non-SMILE regimen (65% vs. 18%, respectively; *P* < 0.001).

### Survival outcomes of advanced-stage ENKL patients

**Fig. 3:**
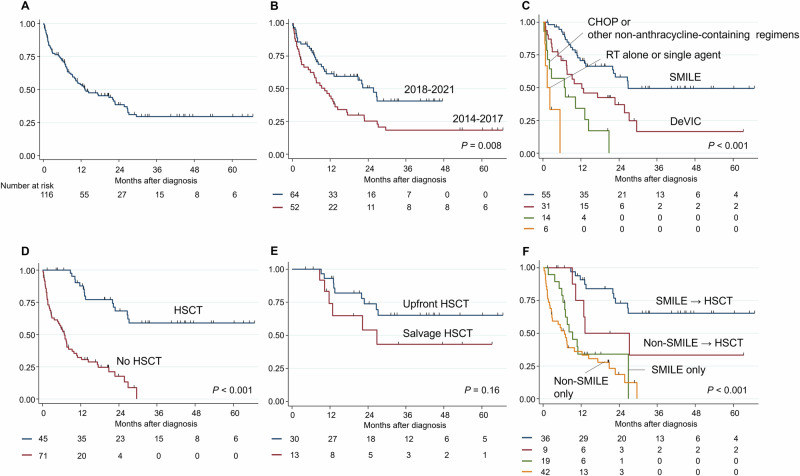
Survival curves of advanced-stage extranodal NK/T-cell lymphoma. **A** The 1-year and 2-year overall survival (OS) of all patients was 53.7% and 38.5%, respectively. The median OS was 13.2 months (95% CI 9.3–22.7). **B** The 2-year OS was 25.2% for patients diagnosed in 2014–2017 and 50.7% for those diagnosed in 2018–2021. **C** The 2-year OS was 58.1% for patients treated with SMILE was 58.1%, but was 37.1% for those with DeVIC, and 0% for those with CHOP/other regimens. **D** The 2-year OS was 68.3% for patients who received HSCT, but was 17.6% for those who did not. **E** The OS of patients who underwent upfront and salvage HSCT was not significantly different (2-year OS: 73.7% vs. 54.0%). **F** The 2-year OS was73.1% for patients treated with SMILE followed by HSCT. In contrast, it was 50.0% for patients treated with SMILE without HSCT, 24.0% for those with non-SMILE regimen and HSCT, and 18.5% for those with non-SMILE regimen without HSCT.

The median follow-up time of survivors was 21 months (range, 0–89 months). The 1-year and 2-year OS of the 116 patients with advanced-stage ENKL was 53.7% and 38.5%, respectively (Fig. 3A). The median OS of the 116 patients was 13.2 months (95% CI 9.3–22.7). In the subgroup analysis, the OS of the advanced-stage ENKL patients has significantly improved in the recent era after 2017 (2-year OS: 25.2% for 2014–2017 vs. 50.7% for 2018–2021: *P* = 0.008; Fig. 3B). Patients aged 60 years or older had a significantly worse prognosis than those younger than 60 years (2-year OS: 46.8% vs. 30.6%: *P* = 0.004; Supplemental Fig. S2). Regarding first-line therapy, patients treated with SMILE had significantly longer OS than those treated with DeVIC, or CHOP/other regimens (2-year OS: 58.1%, 37.1%, and 0%, respectively: *P* < 0.001; Fig. 3C). Forty-five of the 106 treated patients (42%) underwent HSCT, including autologous HSCT (*n* = 19) and allogeneic HSCT (*n* = 26). Patients initially treated with SMILE showed a significantly higher frequency of undergoing HSCT (65%) compared with those treated with DeVIC (23%), or CHOP/other regimens (14%), and RT alone or single agent (0%) (*P* < 0.001). The prognosis was significantly better in patients who underwent HSCT than in those who did not (2-year OS: 68.3% vs. 17.6%: *P* < 0.001; Fig. 3D). Patients who underwent upfront HSCT had better OS than those who did not, although the difference was not statistically significant (2-year OS: 73.7% vs. 54.0%: *P* = 0.16; Fig. 3E). The OS was not significantly different between those who received autologous and allogeneic HSCT (2-year OS: 75.9% vs. 62.4%: *P* = 0.4; Supplementary Figure S3). Regarding response status at HSCT, among 19 patients who underwent autologous HSCT, CR was observed in 15 patients (14 in first CR and 1 in second CR), and PR was observed in 2 patients (both in first PR). Among 26 patients who underwent allogeneic HSCT, CR was observed in 11 patients (all in first CR), and PR was observed in 4 patients (3 in first PR and 1 in second PR). Patients who achieved CR at HSCT tended to have better survival outcomes than those who achieved PR. However, the differences were not statistically significant (Supplementary Fig. S4).

### Central nervous system involvement in advanced-stage ENKL patients

The involvement of the central nervous systems (CNS) was found in 12 patients (10%), including a patient with ocular involvement, at diagnosis (parenchyma, *n* = 5; meninges, *n* = 6; eyeball, *n* = 1). All 12 patients died within 3 years after diagnosis, with a median survival of 7.6 months. The prognosis was significantly worse in the 12 patients with CNS involvement at diagnosis than in those without (2-year OS: 16.7% vs. 41.1%: *P* = 0.03; Supplemental Figure S5).

Among the 104 patients without CNS involvement at diagnosis, 4 (4%) experienced CNS relapse after responding to chemotherapy, with a median time from diagnosis of 14.4 months (range: 9.4–19.9 months), and 1 (1%) did not respond to first-line chemotherapy and further developed CNS involvement. All 4 patients who experienced CNS relapse after responding to chemotherapy were classified as high-risk according to the CNS-PINK. Notably, 2 of the 4 patients had involvement of both adrenal glands at diagnosis. One of these 2 also had the involvement of testis. Another patient had a maximum lesion in the breast with a diameter of 8 cm at diagnosis. Of the 4 patients with CNS relapse after responding to chemotherapy, 2 had CNS involvement only (both meninges), and 2 had systemic disease, with adrenal gland, and CNS involvement (meninges and spinal cord), and with hypopharynx, adrenal gland, and spleen, accompanied by CNS involvement (meninges and spinal cord) at the time of relapse. Two of the 4 patients with CNS relapse initially received SMILE, and the other 2 patients were initially treated with CHOP. One of the 2 patients treated with CHOP did not respond well and was then treated with SMILE as a second-line chemotherapy. Three of the 4 patients responded to chemotherapy and subsequently received HSCT, but they experienced CNS relapse after HSCT. The median OS for the 4 patients with CNS relapse was 5.2 months from the time of CNS relapse (range: 2.9–7.5 months).

### Prognosis of patients treated with SMILE as a first-line treatment

Of the patients initially treated with SMILE who achieved CR or PR (*n* = 39/55, 71%), 29 patients (74%) subsequently underwent HSCT, comprising 25 patients (86%) who underwent upfront HSCT and 4 patients (14%) who underwent salvage HSCT. In contrast, among the patients who were initially treated with SMILE, but did not respond or were not evaluated (*n* = 16/55, 29%), 7 patients subsequently underwent HSCT. Among them, 6 patients (86%) underwent salvage HSCT, and the information regarding the remaining patient was not available. The patients treated with SMILE followed by HSCT had the highest OS compared with those treated with a non-SMILE regimen followed by HSCT, those treated with SMILE without HSCT, and those treated with non-SMILE regimen without HSCT (2-year OS: 73.1%, 50.0%, 34.0%, and 18.5%, respectively: *P* < 0.001; Fig. 3F).

### Prognostic factors affecting overall survival

To identify the prognostic factors affecting the shorter OS of advanced-stage ENKL patients, univariate analysis was performed using Cox’s proportional hazard regression model. Four factors were identified, namely an age of 60 years or older (HR 2.01, 95% CI 1.24–3.25: *P* = 0.005), ECOG-PS of 2 or more (HR 2.60, 95% CI 1.62–4.17: *P* < 0.001), being treated with SMILE (HR 0.33, 95% CI 0.19–0.56: *P* < 0.001), and undergoing HSCT (HR 0.16, 95%CI 0.09–0.30: *P* < 0.001) (Table 2). Multivariate analysis was also performed to control confounding effects, and we finally identified three significant factors: poor ECOG-PS (HR 1.83, 95% CI 1.01–3.30: *P* = 0.045), treatment with SMILE (HR 0.52, 95% CI 0.29–0.96: *P* = 0.04), and undergoing HSCT (HR 0.17, 95%CI 0.08–0.36: *P* < 0.001).

**Table 2:** Univariate and multivariate analyses for overall survival in advanced-stage ENKL patients.

Variable		Univariate analysis			Multivariate analysis		
		HR	(95% CI)	*P*	HR	(95% CI)	*P*
Age	<60	1			1		
	≥60	2.00	(1.24–3.25)	0.005	0.63	(0.32–1.24)	0.18
ECOG-PS	0–1	1			1		
	2–4	2.60	(1.62–4.17)	<0.001	1.83	(1.01–3.30)	0.045
B symptom	Absent	1			1		
	Present	1.61	(0.99–2.61)	0.05	1.55	(0.83–2.91)	0.17
Extranodal involvement	<2	1			1		
	≥2	2.12	(0.97–4.64)	0.06	1.52	(0.68–3.42)	0.3
HSCT	Not done	1			1		
	Done	0.16	(0.09–0.30)	<0.001	0.17	(0.08–0.36)	<0.001
Treatment	Other	1			1		
	SMILE	0.33	(0.19–0.56)	<0.0001	0.52	(0.29–0.96)	0.04

ECOG, Eastern Clinical Oncology Group; PS, performance status; HSCT, hematopoietic stem cell transplantation; HR, hazard ratio; CI, confidence interval.

## Discussion

The present NKEA-Next study demonstrated the latest clinical findings of advanced-stage ENKL patients, including the treatments, prognoses, and trends over the past two decades. The prognosis of advanced-stage ENKL patients has significantly improved in recent years. Patients treated with SMILE had the highest response rate as well as the highest rate of subsequent HSCT. Considering that the patients treated with SMILE and HSCT had the most favorable OS, L-asparaginase-containing chemotherapy followed by upfront HSCT is a promising management strategy for advanced-stage ENKL patients.

More than half of the patients included in this analysis received SMILE chemotherapy as a first-line treatment. The ORR of 71% and CR rate of 49% were almost consistent with those in the previous phase 2 study of SMILE chemotherapy, which corresponded to 79% and 45%, respectively [9]. The efficacy of SMILE for advanced-stage ENKL patients is affirmed in real-world settings. Although the hematologic and nonhematologic AEs were experienced, they were manageable. Therefore, the initial treatment with SMILE resulted in better OS, also after adjusting for confounding factors, such as age, ECOG-PS, and B symptoms, in the multivariate analysis. These results indicate that treatment with an L-asparaginase-containing regimen is beneficial for advanced-stage ENKL patients.

In terms of the trend in treatments for advanced-stage ENKL patients, there has been a notable shift in the use of different therapeutic regimens over time. The proportion of patients treated with CHOP decreased, particularly after 2014, from 22–43% (2000–2013) to 7–12% (2014–2021). DeVIC was the most commonly used for patients diagnosed between 2005 and 2009 (50%), but the proportion of patients treated with DeVIC also decreased afterward (23–45%). In contrast, the proportion of patients treated with SMILE has increased since 2010 and was the highest between 2018 and 2021 (52%). This trend appears to reflect the development of treatments for ENKL patients. Historically, CHOP was commonly employed when effective treatment options were lacking, though its efficacy was unsatisfactory. The development of the DeVIC regimen in the 2000s, originally intended for limited-stage ENKL patients, may have contributed to the increased number of advanced-stage patients treated with DeVIC during that period. Then, since the development of SMILE for advanced-stage patients in 2011, there has been a steady increase in the number of patients receiving SMILE.

The survival outcomes of advanced-stage ENKL patients have improved over the decades. Our present data, encompassing advanced-stage ENKL patients diagnosed between 2014 and 2021, revealed a longer median OS and a higher 2-year OS rate compared with the results from our previous retrospective study that focused on patients diagnosed between 2000 and 2013 [1]. Despite a 2-year OS rate of 38.5%, which remains unsatisfactory, the increasing use of SMILE regimens as first-line chemotherapy suggests an improvement in prognosis. Notably, patients treated with SMILE followed by HSCT exhibited excellent prognoses, with a 2-year OS exceeding 70%. Conversely, those treated solely with SMILE without subsequent HSCT or with non-SMILE chemotherapy (non-L-asparaginase-containing regimen) experienced worse OS rates. Thus, the combination of L-asparaginase-containing regimens and HSCT is crucial for achieving optimal outcomes in advanced-stage ENKL patients. Although our study found no significant difference in prognosis between patients undergoing autologous and allogeneic HSCT, the limited number of patients undergoing HSCT precludes definitive conclusions regarding the superiority of the HSCT type. This warrants further investigation using larger numbers of patients.

The prognosis of ENKL patients after developing CNS involvement was poor. In the present cohort, 4% of the patients without CNS involvement at initial presentation developed CNS relapse, with a median time of 14 months from diagnosis. The CNS relapse rate of ENKL patients was reported as 6.8% in the study from Korea [15], and the median time of CNS relapse from the initial diagnosis was 10 months. The survival duration from CNS relapse was 3.7 months. The study showed that patients who received an intermediate dose of MTX above 2 g/m^2^ had lower CNS relapse rates than those without [15]. Similar to the study from Korea, the incidence of CNS relapse was lower in patients who initially received SMILE (*n* = 2/55, 3.6%) than in patients who initially received CHOP or CHOP-like regimens (*n* = 2/11, 18%) in the present study. In our previous study (NKEA study), 7.5% of the advanced-stage patients experienced CNS relapses, with a median time of 2.6 months after diagnosis [1, 16]. The present study showed low percentage of CNS relapse rates consistent with the previous reports, indicating that it might not be an urgent matter requiring changes in the management of ENKL patients [1, 15, 16].

The limitation of this study is its retrospective nature. The timing of the response evaluation in each patient was not uniform. Although adjustments for potential confounding factors for survival were conducted in the multivariate analyses, they are not complete. Further research that involves a larger cohort of patients with diverse backgrounds and treatment histories is necessary.

Our study revealed the improved prognosis of advanced-stage ENKL patients and highlights the significance of initiating first-line treatment with L-asparaginase-containing chemotherapy followed by HSCT. We believe that our findings will contribute to the enhancement of management strategies for ENKL patients.

## Supplementary information

Supplemental data

## Data Availability

The datasets generated during and/or analyzed during the current study are available from the corresponding author upon reasonable request.
