# Supplementary material for: Improved prognosis of advanced-stage extranodal NK/T-cell lymphoma: results of the NKEA-Next study
Source: Leukemia. 2025 Feb 17;39(4):909–16. doi: 10.1038/s41375-025-02527-4 (PMC11976271; doi:10.1038/s41375-025-02527-4)
Supplement: Supplementary file 1 — Supplemental data [file 41375_2025_2527_MOESM1_ESM.pdf]

Supplemental data

Improved prognosis of advanced-stage extranodal NK/T-cell lymphoma: Results of the NKEA-Next study

Page 2. Supplemental Figures

Page 7. Supplemental Tables

Supplemental Figure Legend

Figure S1 Trend of each treatment between 2000 and 2021.

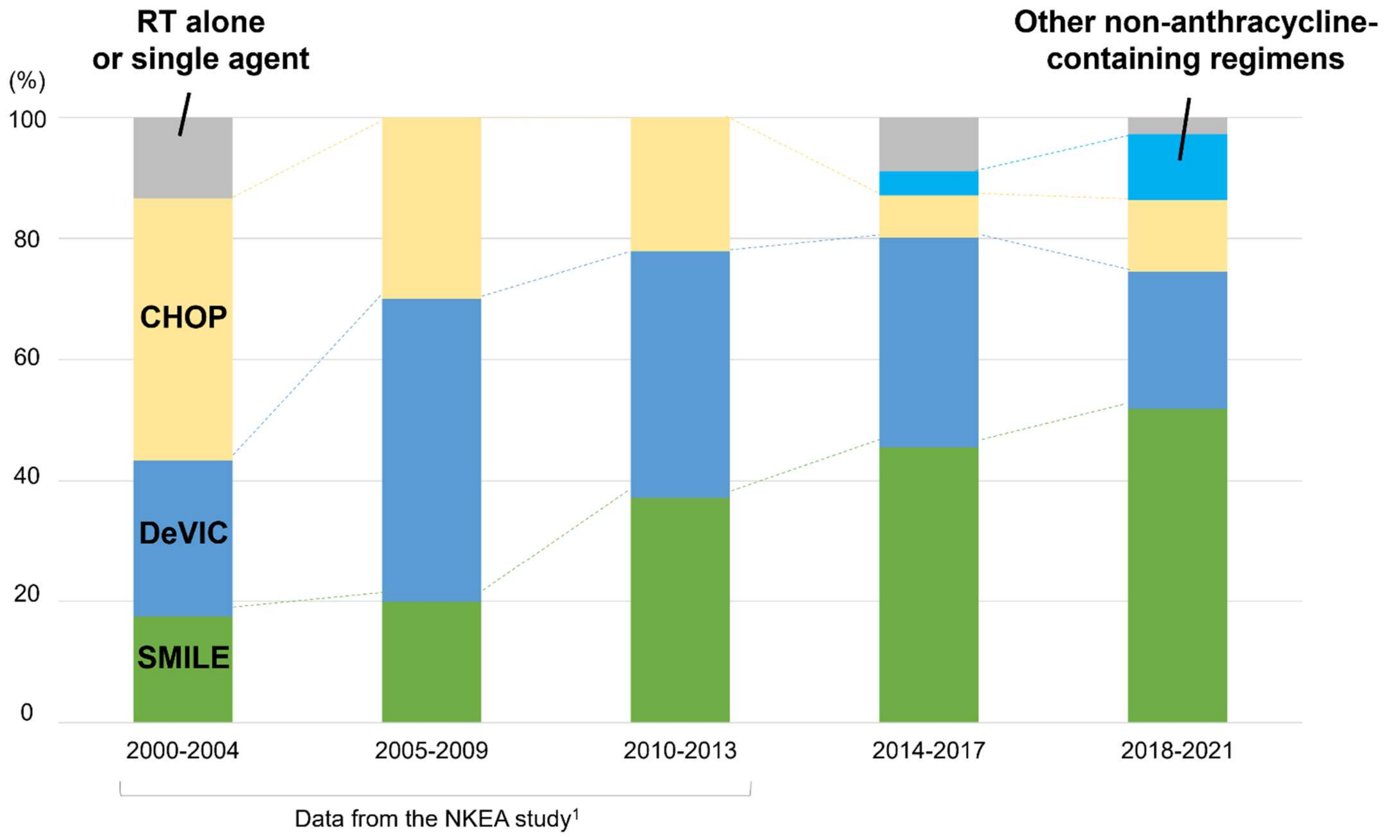

Figure S2

Overall survival of patinets aged 60 years or older and those younger than 60 years.

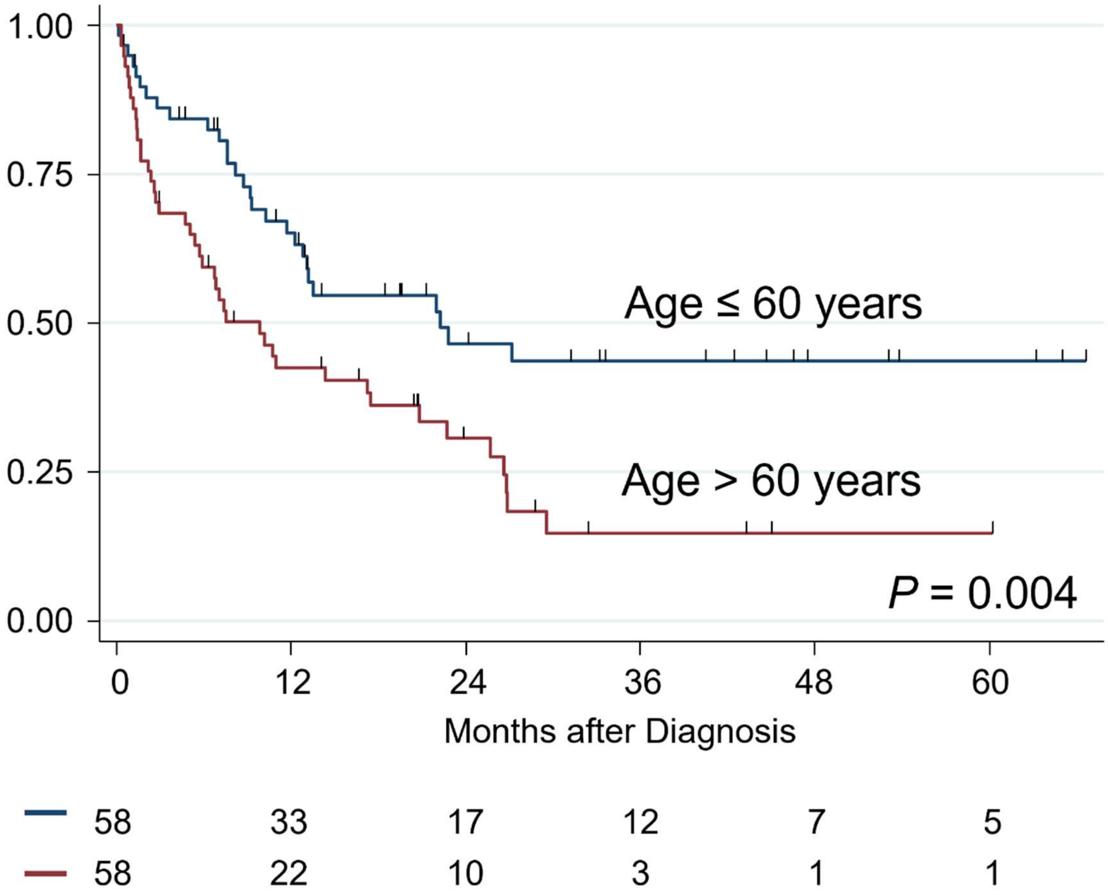

Figure S3

Overall survival of patinets who underwent autologous hematopoietic stem cell translantation (HSCT) and allogeneic HSCT.

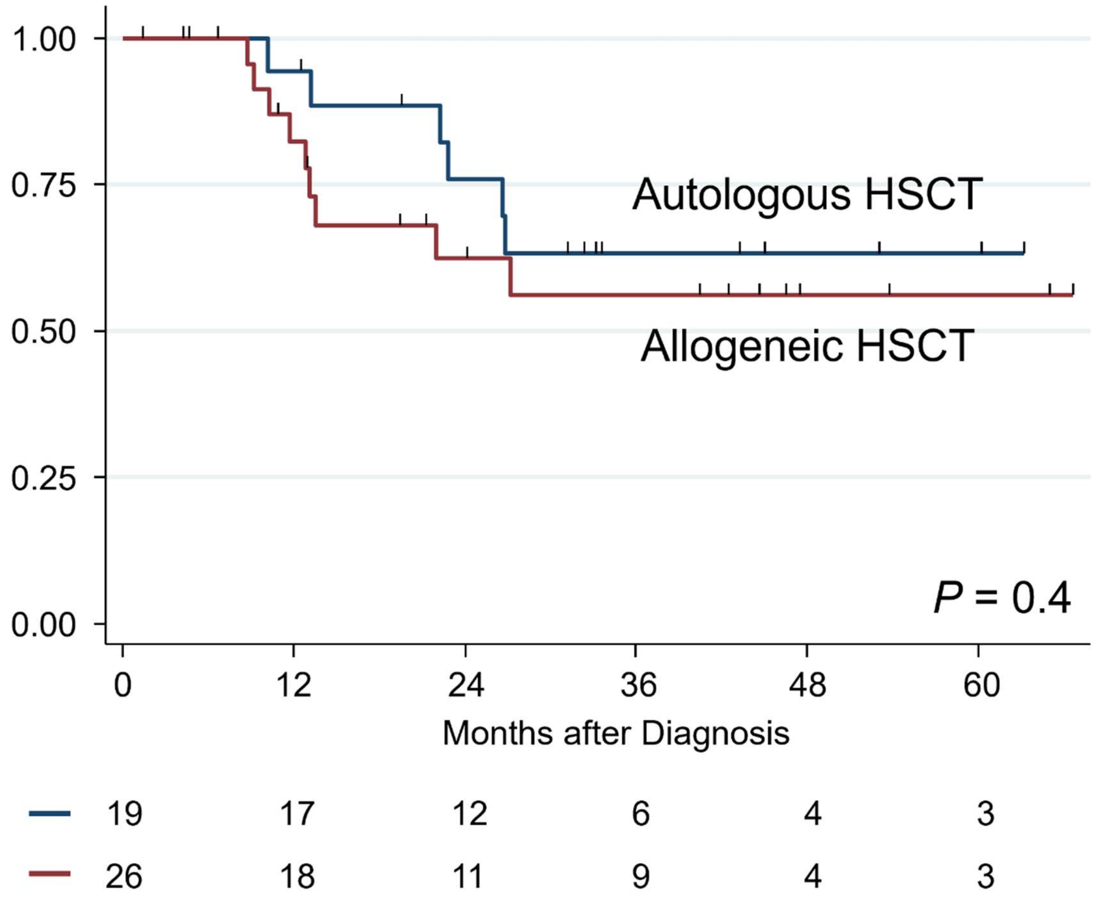

Figure S4

Overall survival of patients who underwent hematopoietic stem cell transplantation (HSCT), stratified by response status at HSCT.

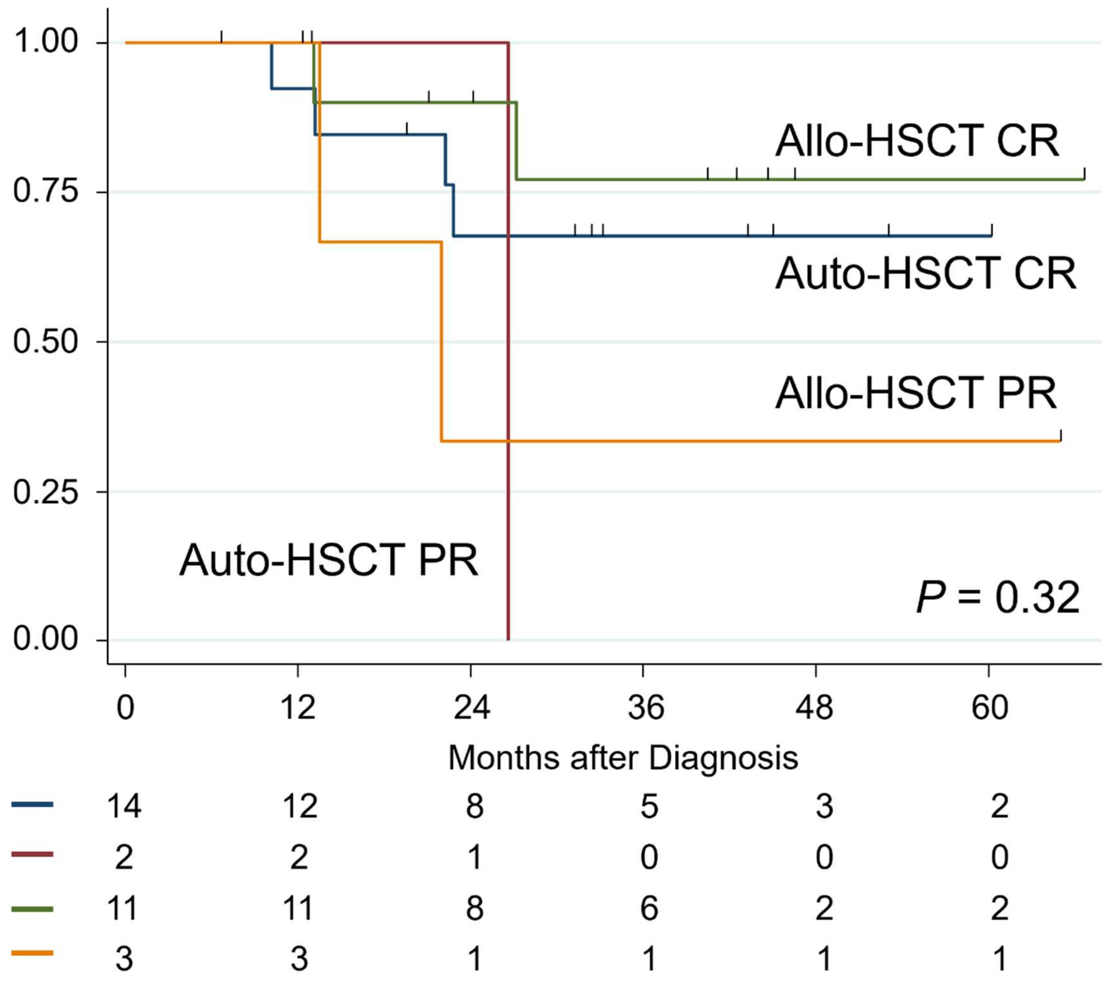

Figure S5

Overall survival of patinets with and without central nervous system (CNS) involvement at the initial diagnosis.

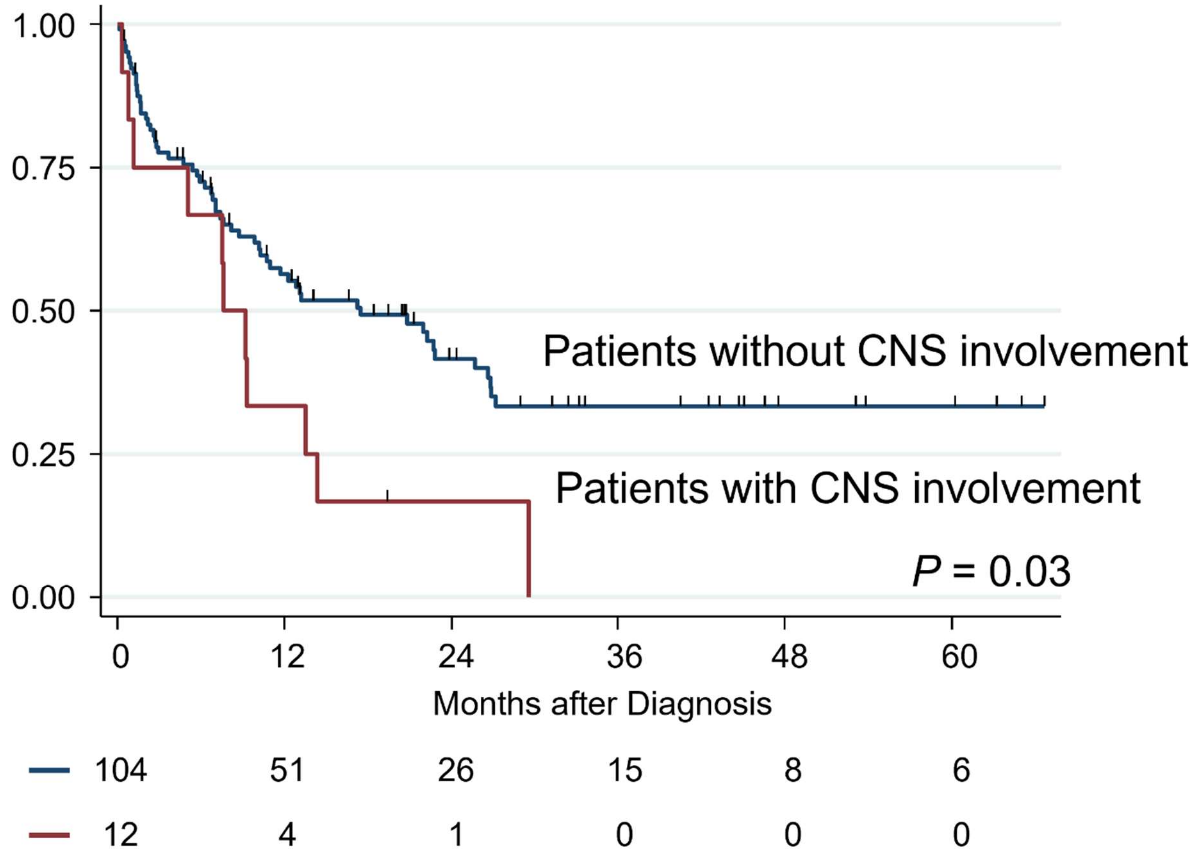

Table S1
Differences of patient characteristics based on HSCT status

**N=116**		**with HSCT (n = 45) N (%)**	**without HSCT (n = 71) N (%)**	*P*
Age	median, years (range)			
	≥ 60 years	7 (16)	51 (72)	< 0.001
Gender	Male	27 (60)	41 (58)	0.85
	Female	18 (40)	30 (42)	
Year at diagnosis	2014-2017	16 (36)	36 (51)	0.08
	2018-2021	29 (64)	35 (49)	
ECOG-PS > 1		10 (22)	30 (43)	0.02
B symptoms		24 (53)	38 (54)	0.54
Stage	III	3 (7)	2 (3)	0.29
	IV	42 (93)	69 (97)	
Extranodal site involvement ≥ 2		35 (81)	59 (87)	0.31
Bulky disease > 7 cm		5 (11)	14 (20)	0.17
Hb < 11 g/dl		3 (7)	25 (35)	<0.001
Plt < 150×10^9 /L		11 (24)	23 (32)	0.24
CRP > 1 mg/dl		18 (40)	32 (45)	0.37
LDH > ULN		33 (73)	40 (56)	0.049
sIL-2R > ULN		38 (84)	60 (87)	0.45
EBV-DNA	Detectable	31 (69)	36 (51)	0.07
	Undetectable	3 (7)	3 (4)	
	Not tested	11 (24)	32 (45)	
PINK	Intermediate (1)	14 (31)	6 (8)	0.002
	High (2-4)	31 (69)	65 (92)	
PINK-E	Low (1)	1 (2)	1 (1)	0.048
	Intermediate (2)	12 (27)	6 (8)	
	High (3-5)	21 (47)	33 (46)	
	Not evaluable	11 (24)	31 (44)	

Abbreviations: ECOG, Eastern Clinical Oncology Group; PS, performance status; Hb, hemoglobin; PLT, platelet; CRP, C-reactive protein; LDH, lactate dehydrogenase; ULN, upper limit of normal; sIL-2R, soluble interleukin-2 receptor; EBV, Epstein-Barr virus; PINK, prognostic index of natural killer lymphoma; HSCT, hematopoietic stem cell transplantation.

*Only patients with EBV-DNA detectable in the peripheral blood

Table S2
First-line treatment of advanced-stage ENKL patients

Treatment	N (%)
**i) Chemotherapy alone**	**86 (74)**
SMILE	51 (44)
DeVIC*	21 (18)
CHOP	10 (9)
EPOCH	1 (1)
GDP	1 (1)
L-asp or CyA alone	2 (2)
**ii) Concurrent CRT**	**7 (6)**
RT-DeVIC*	6 (5)
RT-GCD	1 (1)
**iii) Sequential CRT**	**9 (8)**
DeVIC	4 (3)
SMILE	4 (3)
HD-MTX	1 (1)
**iv) RT alone**	**4 (3)**
**v) None**	**10 (9)**

Abbreviations: SMILE, steroid, methotrexate, ifosfamide, L-asparaginase, and etoposide; DeVIC, dexamethasone, etoposide, ifosfamide, and carboplatin; CHOP, cyclophosphamide, doxorubicin, vincristine, and prednisone; EPOCH, etoposide, cyclophosphamide, doxorubicin, vincristine, and prednisone; GDP, gemcitabine, dexamethasone, and cisplatin; L-asp, L-asparaginase; GCD, gemcitabine, carboplatin, and dexamethasone; CyA, cyclosporine A; HD-MTX, high-dose methotrexate; RT, radiotherapy.

*One patient from each group received chemotherapy with L-asparaginase.

Table S3
Severe adverse events of grade 3 or higher for ENKL patients treated with SMILE.

Adverse event	Grade 3		Grade 4		Grade5	
	N	(%)	N	(%)	N	(%)
Febrile neutropenia	7	(13)	0	(0)	0	(0)
Liver dysfunction	6	(11)	0	(0)	0	(0)
Anorexia	4	(7)	0	(0)	0	(0)
Mucositis oral*	4	(7)	0	(0)	0	(0)
Blood bilirubin increased	2	(4)	0	(0)	0	(0)
Sepsis	2	(4)	3	(6)	0	(0)
Lung infection	1	(2)	0	(0)	0	(0)
Enterocolitis infectious	1	(2)	0	(0)	0	(0)
Anaphylaxis	1	(2)	1	(2)	0	(0)
Allergic reaction	1	(2)	0	(0)	0	(0)
Pancreatitis	1	(2)	0	(0)	0	(0)
Serum amylase increased	1	(2)	0	(0)	0	(0)
Fibrinogen decreased	1	(2)	0	(0)	0	(0)
Lipase increased	0	(0)	1	(2)	0	(0)
Acute kidney injury	0	(0)	1	(2)	0	(0)
Gastric hemorrhage	0	(0)	1	(2)	0	(0)
Dyspnea	0	(0)	0	(0)	0	(0)
Interstitial pneumonitis	0	(0)	0	(0)	1	(2)

Abbreviations: AST, aspartate aminotransferase; ALT, alanine transaminase; GGT, gamma-glutamyl transferase.

* One of the 4 patients who developed oral mucositis received SMILE after RT.
